# Characterization of a Wheat Heme Oxygenase-1 Gene and Its Responses to Different Abiotic Stresses

**DOI:** 10.3390/ijms12117692

**Published:** 2011-11-08

**Authors:** Dao-kun Xu, Qi-jiang Jin, Yan-jie Xie, Ya-hui Liu, Yu-ting Lin, Wen-biao Shen, Yi-jun Zhou

**Affiliations:** 1 College of Life Sciences, Cooperative Demonstration Laboratory of Centrifuge Technique, Nanjing Agricultural University, Nanjing 210095, China; E-Mails: 2009116134@njau.edu.cn (D.X.); 2010216030@njau.edu.cn (Q.J.); 2008216036@njau.edu.cn (Y.X.); webmasterone@sina.com (Y.-h.L.) 2010116133@njau.edu.cn (Y.-t.L.); 2 Institute of Plant Protection, Jiangsu Academy of Agricultural Sciences, Nanjing 210014, Jiangsu, China

**Keywords:** abiotic stress responses, carbon monoxide, heme oxygenase-1, prokaryotic expression in *E. coli*, wheat

## Abstract

In animals and recently in plants, heme oxygenase-1 (HO1) has been found to confer protection against a variety of oxidant-induced cell and tissue injuries. In this study, a wheat (*Triticum aestivum*) HO1 gene *TaHO1* was cloned and sequenced. It encodes a polypeptide of 31.7 kD with a putative *N*-terminal plastid transit peptide. The amino acid sequence of TaHO1 was found to be 78% similar to that of maize HO1. Phylogenetic analysis revealed that TaHO1 clusters together with the HO1-like sequences in plants. The purified recombinant TaHO1 protein expressed in *Escherichia coli* was active in the conversion of heme to biliverdin IXa (BV), and showed that the *V*_max_ was 8.8 U·mg^−1^ protein with an apparent *K_m_* value for hemin of 3.04 μM. The optimum Tm and pH were 35 °C and 7.4, respectively. The result of subcellular localization of TaHO1 showed that the putative transit peptide was sufficient for green fluorescent protein (GFP) to localize in chloroplast and implied that TaHO1 gene product is at least localized in the chloroplast. Moreover, we found that *TaHO1* mRNA could be differentially induced by the well-known nitric oxide (NO) donor sodium nitroprusside (SNP), gibberellin acid (GA), abscisic acid (ABA), hydrogen peroxide (H_2_O_2_) and NaCl treatments. Therefore, the results suggested that *TaHO1* might play an important role in abiotic stress responses.

## 1. Introduction

A wide range of environmental stresses, such as salinity, drought, UV, and heavy metals exposure, are potentially harmful to plants. A common aspect to all of these adverse conditions is the enhanced production of reactive oxygen species (ROS) within several subcellular compartments of the plant cells [1]. In order to cope with above abiotic stresses, some antioxidative systems such as superoxide dismutase (SOD), catalase (CAT), and ascorbic acid peroxidase (APX) are proved responsible for the protection against toxic ROS [2]. Recently, another system, heme oxygenase-1 (HO1), which belonged to the inducible and major isoform of heme oxygenases (HOs; EC 1.14.99.3), gained more attention in both animals and plants because of its assumed responsibility for the detoxification of ROS and free radicals [3]. It was well established that HO could catalyze the oxidation of heme with molecular oxygen, which results in the formation of biliverdin-IXα (BV), carbon monoxide (CO) and free iron [4–7]. In mammals, BV and its reduced derivatives, bilirubin (BR), have strong antioxidant properties both *in vitro* and *in vivo* [4,8], thus resulting in the speculation that HO1 might have a protective role against tissue injury [9].

In Arabidopsis, HOs include a small gene family with four members in total which can be categorized into two subfamilies, HO1 and HO2 [10,11]. It is known that HO1 family includes three isoforms: HY1, HO3, and HO4, while the HO2 family only has one member: HO2. All the four members of the HO family are transcriptionally active with substantially overlapping patterns of expression. Some evidence also showed that HO1 is clearly the most highly expressed, followed by HO2, while both HO3 and HO4 expressed at low levels [10]. In other plants, HO1 has been proved to be an inducible enzyme and can be induced by multiple stimuli and various abiotic stresses, including its own substrate heme [12], heavy metals [13,14], glutathione depletion [15], UV radiation [16], salinity stress [17–19], and H_2_O_2_ [20]. These responses were thus thought to be a cellular defense mechanism against various stresses-triggered oxidative damage and also exhibit hormone-like bioactivity [3,21].

Until now, the researches on plant HO1 genes have only focused on a few plant species such as Arabidopsis (*AtHO1*, also called *HY1*) [22], rice (OsHO-1, also called *SE5*) [23], alfalfa (*MsHO1*) [12], and Chinese cabbage (*BrHO1*) [24], *etc*. Wheat (*Triticum aestivum*) is one of the most important crops worldwide. To date, the characterization of wheat HO1 gene remained unknown because of its limited genomic information [19]. However, analysis of wheat expressed sequence tags (ESTs) generated from various tissues during plant development or upon different stress conditions gives insights into the transcribed portions of the genome. In this test, to get first insight into the role of wheat HO1, we reported on molecular cloning and characterization of a HO1 cDNA from wheat (named as *TaHO1*). Sequence analyses of the deduced amino acids of its encoded protein were analyzed and their phylogenetic relationships were compared with other HOs from various plant species. Furthermore, subcellular localization of TaHO1 protein was surveyed. We further purified recombinant mature TaHO1 protein expressed from an *Escherichia coli* expression system and detected its HO enzyme activity successfully, then characterized this protein in terms of spectroscopic and catalytic properties. The expression patterns of *TaHO1* by real-time RT-PCR were investigated in various wheat seedling tissues under the normal growth conditions and with different exogenous chemicals and salinity stress. Therefore, the characterization of *TaHO1* will provide insight into the physiological processes of stress responses in wheat plants.

## 2. Results and Discussion

### 2.1. Identification and Cloning of a Wheat HO1 Gene

Abundant *ESTs* in public databases provide a source for the identification of new genes and for comparative analyses among different organisms. In this study, we searched the wheat EST database in NCBI with the maize *HO1* sequence. This search obtained several EST sequences with high homology to the query, and a putative wheat *HO1* gene was assembled by ORF analysis. One full length cDNA of 867 bp was amplified from wheat seedling leaves with primer pairs P1 designed based on this assembled *HO1*. We noticed that the predicted protein product of its sequence comprises 288 animo acids which shared 99% identity with the deduced product of the assembled wheat *HO1* sequence. Furthermore, we discovered that this sequence was identical to the maize *HO1* and designated as *TaHO1*. For example, the nucleotide sequence showed the highest similarity (60%) to that of the maize *HO1* gene, within the predicted coding region and the putative translated product was 78% identical. The predicted molecular mass of TaHO1 was 31.7 kD and its theoretical isoelectric point was 6.41. The sequence of this cDNA was deposited in GenBank with the accession ID HM014348.

**Figure 1:**
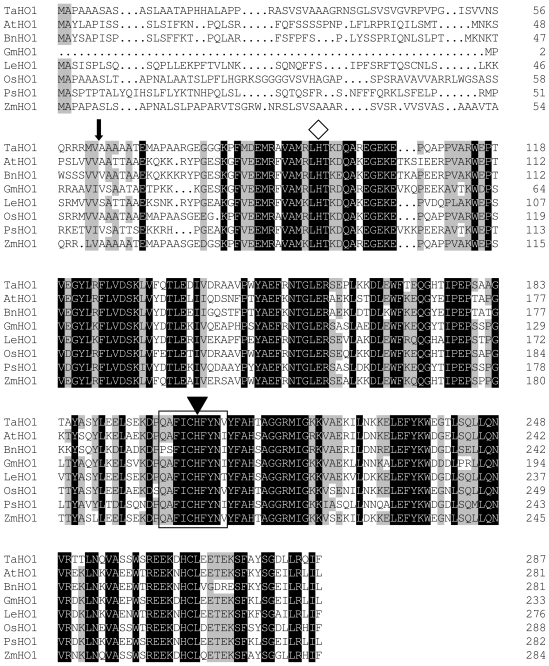
The wheat *HO1* gene. Alignment of TaHO1 with other plant HO1s. Dark shading with white letters and gray shading with black letters reveal 100% and 75% sequence conservation, respectively. Database accession numbers are ADG56719 for TaHO1 (*Triticum aestivum*), AB021858 for AtHO1 (*Arabidopsis thaliana*), GU390397 for BnHO1 (*Brassica napus*), AF320024 for GmHO1 (*Glycine max*), AF320028 for LeHO1 (*Lycopersicon esculentum*), EU781632 for OsHO1 (*Oryza sativa*), AF276228 for PsHO1 (*Pisum sativum*), and EU962994 for *Z*mHO1 (*Zea mays*), respectively. Solid frame means signature sequence. Also, the arrow identified the predicated cleavage site between the transit peptide and mature protein. The conserved histidine residue involved in heme-iron binding and catalysis was shown by a white diamond. The conserved histidine residue for protein stability was illustrated by a reverse triangle.

Like that of the Arabidopsis counterpart HY1 [22], computer analysis by the ChloroP program predicted that TaHO1 might also localize into the chloroplast, and its predicted chloroplast transit peptide cleavage site is most likely positioned between amino acids 62 and 63 (Figure 1). The alignment of the amino acid sequences of wheat and other HO1 further illustrated that a total of 52.7% of residues is conserved, and almost all amino acids are conserved in the HO signature sequence (QAFICHFYNI/V) which corresponds to Q199–V208 in TaHO1 (Figure 1, boxed). The signature sequence was also conserved in mammalian HO1 sequences [5]. Interestingly, Arabidopsis HY1 contains four histidine residues and all of them are conserved in other plant HO1. In fact, although the amino acid sequences of these plant HO1 proteins are not closely related to mammalian HO sequences [22], His-132 in rat HO-1, which is thought to play a structural role in stabilizing the HO protein [25], is maintained in TaHO1 as His 204. The proximal heme-binding ligand of human HO1, His25 [26], is also conserved in TaHO1 as His95. Previous report demonstrated that in the first step of the reaction of HO, heme bound to an amino acid residue of the HO protein through the fifth co-ordination portion of the iron atom to form an enzyme-substrate complex, and that the absorption spectrum of the complex formed closely resembled that of hemoglobin and myoglobin [27]. Therefore, some of these histidine residues, conserved in those sequences, are probably important for heme binding. Together, all these data clearly suggested that this cDNA encodes HO.

### 2.2. Phylogenetic Analysis of TaHO1

**Figure 2:**
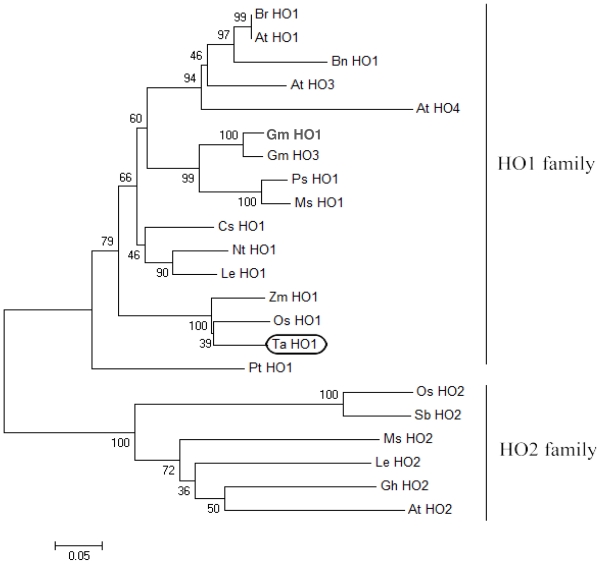
The phylogenetic relationships of mature HO1/2 isoforms in plants. The protein sequence for the mature region of TaHO1 was aligned with mature sequences of previously identified plant HO proteins. Besides shown in Figure 1, other database accession numbers are as follows: *Brassica campestris* (BrHO1, HQ690823), *Arabidopsis thaliana* (AtHO2, AAD22109; AtHO3, NP177130; AtHO4, AAK63007), *Glycine max* (GmHO3, AAK63009), *Medicago sativa* (MsHO1, ADK12637; MsHO2, HQ652868), *Cucmis sativus* (CsHO1, ADO08223), *Nicotiana tabacum* (NtHO1, HQ690822), *Lycopersicon esculentum* (LeHO2, AF320029.1), *Oryza sativa* (OsHO2, BM293897.1), *Pinus taeda* (PtHO1, AAK63014), *Sorghum bicolor* (SbHO2, AAK63011) and *Gossypium hirsutum* (GhHO2, EU373020). Predicted *N*-terminal chloroplast transit peptide domains were removed based on predictions using ChloroP. Sequences were aligned by CLUSTALW and analyzed using the MEGA 4.1. Construction was carried out by the parsimony method with 1000 bootstrap replicates and a consensus tree was generated.

Other plant HOs discovered in GenBank were used for phylogenetic analysis to identify the evolutionary relationships among TaHO1 and other HOs (Figure 2). The *N*-terminal transit peptides of plant HOs were neglected in the analysis because this region was highly variable and not subject to the same evolutionary constraints as the regions encoding the mature protein. The phylogenetic tree showed that the plant HOs can be separated into two main divisions, HO1-like and HO2-like sequences. The TaHO1 was clearly grouped with the HO1-like sequences supporting its designation as *TaHO1*. Normally, plant HO1s group into a number of families based upon established taxonomic divisions. Consistent with this, *TaHO1* clearly groups with other sequences from the *Poaceae*, such as *Zea mays* and *Oryza sativa* (especially). Additionally, we found several HO1-like sequences existing in each species such as Arabidopsis (HO1, HO3, and HO4) while only one HO2-like sequence appeared. This indicates that the divergence of the two isoforms occurred before the speciation of these plants, and these HO1s might be a result of gene duplication of an ancestral copy of HO1 following the speciation.

### 2.3. Expression and Purification of TaHO1

**Figure 3:**
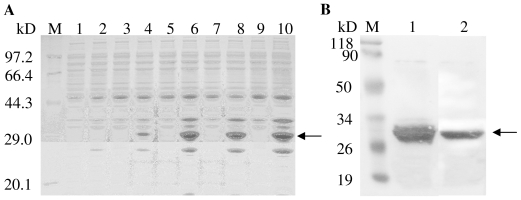
Expression and analysis of the recombinant *His*-tagged mTaHO1 protein in *E. coli*. (**A**) Expressed proteins were induced with IPTG, 30 μg protein/well. Lane M, Marker proteins; lane 1, protein from IPTG-induced bacteria for 12 h harboring pET-28a(+) alone as control; lane 2,3,5,7, and 9, proteins from uninduced bacteria for 0, 1, 3, 6 and 12 h, respectively; lane 4,6,8 and 10, proteins from IPTG-induced bacteria for 1, 3, 6 and 12 h, respectively; (**B**) Expressed protein purified by Ni-affinity chromatography and its gel blot analysis, 60 μg protein/well. Lane M, Marker proteins; lane 1, purified protein; lane 2, immunoblot of purified protein developed with the polyclonal antiserum against the mature wheat HO-1 (mTaHO1) with a molecular mass of 31 kDa.

To confirm that TaHO1 encodes a HO and to further characterize its properties, we expressed the mature TaHO1 (mTaHO1) (without the predicted signal peptides) as the *N*-terminal *His*-tagged recombinant protein in *E. coli* by using the pET-28a(+) vector. The mature recombinant TaHO1 induced by IPTG was expressed as a soluble protein with the intensity in a time-dependent mode and formed a 31 kD band (Figure 3A, lane 4,6,8,10), approximately corresponding to the molecular weight of the mTaHO1 protein (25.6 kD) plus that of 6× *his*-tag (0.8 kD) and the translated vector sequence (4 kD). Whereas, *E. coli.* cells transformed with the empty vector did not contain proteins in this mass region (Figure 3A, lane 1). Furthermore, the purification of TaHO1 by Ni-affinity chromatography yielded a protein that formed a similar band on SDS-PAGE (Figure 3B, lane 1) and exhibited immunoreactivity with its specific antibody (Figure 3B, lane 2).

### 2.4. Biochemical Activity of the Mature TaHO1 Protein Expressed in Escherichia coli

**Figure 4:**
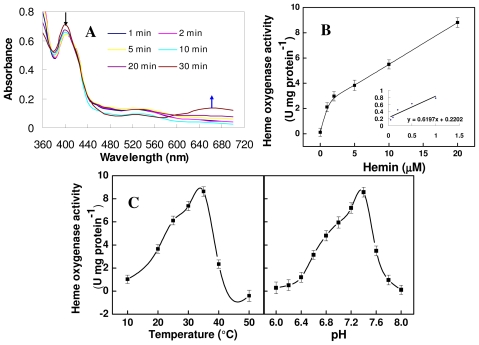
Biochemical characterization of purified recombinant mTaHO1. (**A**) Time-dependent absorbance changes were determined during the mTaHO1 reaction with spectra between 360 and 700 nm taken at 1, 2, 5, 10, 20 and 30 min after the addition of NADPH. The black arrow indicates the degradation of hemin and the blue arrow stands for the generation of BV; (**B**) Michaelis-Menten plot of the mTaHO1 reaction for hemin concentrations of 1, 2, 5, 10 and 20 μM. Insert: Lineweaver-Burk plot of the same data; (**C**) Temperature (left) and pH (right) dependence of the mTaHO1 reaction. Data shown are the means ± SD from three independent measurements.

Furthermore, the purified recombinant protein was tested for heme ring cleavage activity by following the conversion of heme to BV spectrophotometrically. As shown in (Figure 4A), TaHO1 showed similar catalytic characteristics in heme cleavage as previously characterized HY1 in Arabidopsis [28], and BrHO1 in Chinese cabbage [24]. The enzyme first forms a complex with heme, which can be monitored photometrically and results in a maximum absorbance of the heme-TaHO1 complex at 405 nm (Figure 4A). Over a period of 30 min, the reaction proceeded with a decrease in 405 nm, since the complex was converted to a ferric biliverdin-HemT complex and subsequently to free biliverdin, as was apparent from the formation of a broad peak at approximate 660 nm. These results indicated that this purified TaHO1 protein was able to degrade heme.

The plot of HO activity versus hemin concentration showed normal Michaelis-Menten kinetics (Figure 4B) and the kinetic constants were obtained from a Lineweaver-Burk plot (Figure 4B, insert). Under our experimental conditions, the *V*_max_ was 8.8 U·mg^−1^ protein with an apparent *K*_m_ value for hemin of 3.04 μM. Comparable results were observed in previous analyses for human HO1 [29], Arabidopsis HY1 [28], alfalfa MsHO1 [12], and Chinese cabbage BrHO1 [24]. Additionally, the optimum Tm was 35 °C (Figure 4C), which is different from the values of 50 °C reported for recombinant Arabidopsis HY1 [28], and dropped sharply either side of this value. Meanwhile, pH dependence of the mTaHO1 reaction showed that the rate of the mTaHO1 reaction increased to a peak value at pH 7.4 and declined thereafter.

### 2.5. Subcellular Localization of Green Fluorescent Protein (GFP) Fusion Protein

**Figure 5:**
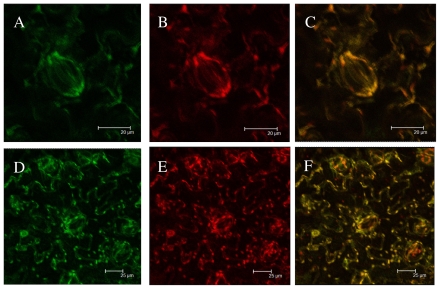
Subcellular localization of TaHO1 by transient expression of the fused fluorescent proteins. Microscopic images of GFP (**A** and **D**), chloroplast autofluorescence (**B** and **E**), and merge (**C** and **F**) from tobacco epidermal cells infected with *Agrobacteria* harboring the GFP constructs. GFP fused to TaHO1 transit peptide (**A**–**C**, scale bar = 20 μm) and GFP alone (negative control, **D**–**F**, scale bar = 25 μm). The photographs were taken in the blue channel (**A** and **D**), red channel (**B** and **E**), or their combination (**C** and **F**).

To demonstrate that predicted chloroplast targeting sequences of TaHO1 are able to sort the protein into chloroplast, a vector was made with a GFP fusion construct containing the predicted chloroplast transit peptide of TaHO1 upstream from GFP. After transfection of tobacco leave cells with EHA105 containing this construct, GFP was detected using confocal laser scanning microscopy in subcellular compartments with oval structure proven to be chloroplast as demonstrated by the overlap of red autofluorescence from the chlorophyll of the chloroplasts and the GFP fluorescence in it (Figure 5). The control construct without the putative transit peptide showed GFP fluorescence throughout the plasma membrane of tobacco leaves. This suggests the transit peptide of TaHO1 is functional and sufficient to transport TaHO1 to plastids, further implying that the *TaHO1* gene product in wheat plants is most likely localized in the plastids.

### 2.6. Expression pattern Analysis of TaHO1 Protein

**Figure 6:**
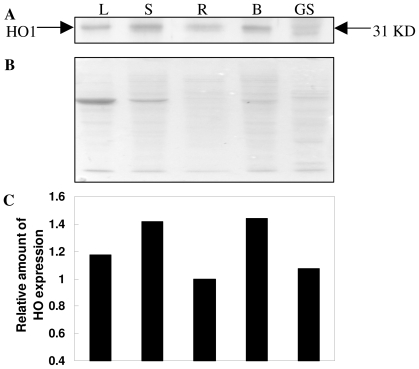
TaHO1 protein levels in various wheat tissues. Young leaves (L), stems (S), roots (R), and buds (B) were collected from 14-d-old seedlings under the normal growth conditions. Meanwhile, germinating seeds (GS) were sampled after 3 d of germination. Then, TaHO1 protein levels were determined by protein gel blot analysis (**A**); Meanwhile, Coomassie Brilliant Blue-stained gels (**B**) were present to show that equal amounts of proteins were loaded. Densitometry was done by Quantity One software to quantify HO-1 protein level (**C**). Relative HO-1 protein level taking root sample (R) as 100%.

The tissue-specific expression patterns of the TaHO1 protein in wheat seedlings were analyzed by gel blot analysis. The results of Figure 6 showed that TaHO1 protein levels were expressed in wheat leaves, stems, roots, and buds. It was also found that TaHO1 protein was more accumulated in stems and buds, but in roots with low levels (Figure 6). We also noticed that in germinating wheat seeds, two bands with a molecular mass of about 31 kD appeared apparently, both of which were recognized by the rabbit polyclonal antibody against the TaHO1 protein. These suggested that another HO isoform as well as TaHO1, may play essential roles in wheat seed germination process.

### 2.7. Expression of TaHO1 Gene in Responses to Different Exogenous Chemicals and Salinity Stress

**Figure 7:**
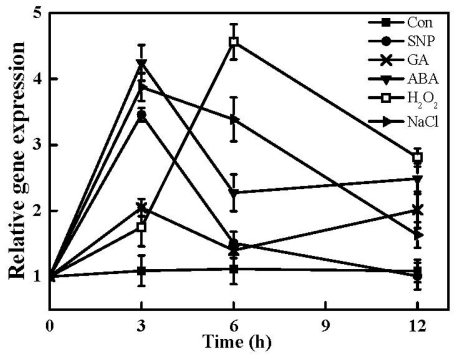
Induction of *TaHO1* in response to SNP, GA, ABA, H_2_O_2_, and NaCl treatments. Real-time RT-PCR was carried out from the total RNA isolated from the second leaves of wheat seedlings treated with 100 μM SNP, 50 μM GA, 100 μM ABA, 100 μM H_2_O_2_ or 100 mM NaCl at the indicated time points. Relative expression levels of *TaHO1* were presented relative to those of corresponding samples at 0 h, respectively.

Recently, the HO1/CO system in plants has attracted more interest due to its physiological cytoprotective roles. For example, biological functions of HO-1 protein or corresponding genes are believed to be associated with a fundamental adaptive and defensive responses against osmotic and salinity stresses [17,18,30], UV irradiation [16], and heavy metal exposure [13,14]. In the subsequent test, the expression profiles of the *TaHO1* gene in wheat seedling leaves treated with different exogenous chemicals and NaCl were analyzed by real-time RT-PCR. Time-course analysis of gene expression showed that *TaHO1* transcripts could be differentially induced by SNP (sodium nitroprusside), GA, ABA, H_2_O_2_ and NaCl treatments (Figure 7). For example, *TaHO1* transcripts were up-regulated sharply by NaCl and SNP while gradually by H_2_O_2_ during the first 3 h treatment period then decreased except treated with H_2_O_2_. Meanwhile, a biphasic burst of *TaHO1* gene expression was appeared in GA treatment. The time course experiments illustrated that a fast increment of *TaHO1* transcripts appeared as early as 3 h after GA treatment, followed by a gradual decrease until reaching another peak until 12 h.

NO in the plant kingdoms has been suggested to act as a signal molecule mediating various responses to biotic and abiotic stresses, including salinity and osmotic stresses as well as UV irradiation [31]. Thus, the sharp enhancement of *TaHO1* transcripts induced by the NO donor SNP during the early stage of treatment indicated that *TaHO1* might be involved in plant tolerance against biotic and abiotic stresses through NO signaling pathway. Previously, studies showed that *HO1* gene from other plants could be induced by some agents or stimuli, including its own substrate heme [12], salinity stress [17,18], glutathione depletion [15], and H_2_O_2_ [20]. A common characteristic of these inducers is their ability to cause oxidative stress [3]. The results of Genevestigator DNA microarray database [32] showed that the *HY1* mRNA was induced significantly in Arabidopsis seedlings upon osmotic and salinity stresses. Our recent study further showed that *HY1* overexpression plant lines exhibited an enhanced salt tolerance [18]. Moreover, HO1 in soybean plants has been found to be induced significantly by UV-B irradiation [16] and salinity stress [33]. Although precisely how the expression of *HO1* is induced by these agents remains to be determined, in most cases, the induction has been confirmed to be associated with the alleviation of the oxidative stress.

Thus, above findings of *TaHO1* suggested that the members of the HO1 isoform in plants are not only structurally similar, but also share a conserved role in plant responses against oxidative stresses [3]. Our results of responsiveness of *TaHO1* gene to SNP, GA, ABA, H_2_O_2_ and NaCl also provide a possible link between the HO1 and the maintenance of cellular homeostasis.

## 3. Experimental Section

### 3.1. Plant Materials, Growth Condition and Treatments

All chemicals were obtained from Sigma (St Louis, MO, USA) unless stated otherwise. Commercially available wheat (*Triticum aestivum*, Yangmai 13) seeds were surface-sterilized with 5% NaClO for 10 min, rinsed extensively in distilled water and germinated for 1 d at 25 °C in the darkness. Then, uniform buds were chosen and carefully transferred to the plastic chambers and cultured in modified Hoagland solution [17]. Wheat seedlings were grown in the illuminating incubator at 25 ± 1 °C, with a light intensity of 300 μmol·m^−2^·s^−1^ and 12 h photoperiod. The culture solution was renewed every other day until two fully expanded leaves appeared. Then, wheat seedlings were incubated in modified Hoagland solution containing 100 μM sodium nitroprusside (SNP), the well-known nitric oxide (NO) donor, 50 μM GA, 100 μM ABA, 100 μM H_2_O_2_ or 100 mM NaCl. The second fully expanded leaves were harvested at 0, 3, 6 and 12 h of various treatments. For tissue-specific expression analysis, young leaves (L), stems (S), roots (R), and buds (B) were collected from 14-d-old seedlings under the normal growth conditions. Meanwhile, germinating seeds (GS) were sampled after 3 d of germination. All harvested samples were immediately used or frozen in liquid nitrogen, and then stored at −80 °C until further analysis. Three different experiments were performed, with at least three replicated measurements for each parameter assayed.

### 3.2. Cloning of TaHO1

Blast [34] searches were employed to search wheat ESTs in the dbEST databases that were homologous with the mRNA sequence of the maize full-length *HO1* gene (GenBank ID: NM_001199033). A putative wheat *HO1* gene sequence was assembled by comparative alignments of corresponding EST sequences. The specific sequences were used to design PCR primer pairs P1 (F: 5′-TCCGGATCCATGGCGCCCGCGGCAGC-3′ and R: 5′-GTTAAGCTTTTAGGTGAATATCTGGCGTAG-3′), which would amplify the entire coding region of wheat *HO1* cDNA by RT-PCR.

Total RNA was extracted from wheat seedling leaves using Trizol reagent (Invitrogen, Gaithersburg, MD) according to the manufacturer’s instructions. First-strand cDNAs were synthesized from 2 μg total RNA (pre-treated with DNase I) with AMV reverse transcriptase (TaKaRa) according to the manufacturer’s protocol. The PCR conditions for amplifying TaHO1 were as follows: 10 min at 94 °C; 32 cycles of 30 s at 94 °C, 50 s at 50 °C, 1 min at 72 °C; and a final extension for 10 min at 72 °C. The PCR product was gel-purified and cloned into pMD-19T vector (TaKaRa) for sequencing (GenScript, Nanjing, China).

### 3.3. Multiple Sequence Alignment and Phylogenetic Analysis

Alignment of deduced TaHO1 or other plant HO protein sequences were performed with DNAMAN 6.0.40 software. The phylogenetic tree of mature plant HO proteins was constructed with MEGA program (ver 4.1) by neighbor-joining (NJ) method. The parameters pairwise deletion and p-distance model were used. Bootstrap test of phylogeny was performed with 1000 replicates.

### 3.4. Prokaryotic Expression and Purification of Recombinant *His*-Tagged TaHO1

A mature TaHO1 (mTaHO1) without the predicted chloroplast transit peptide was subcloned into the *Escherichia coli* expression vector pET-28a (+) (Novagen) to produce pET-28a(+)-mTaHO1. mTaHO1 was amplified using the primer pairs P2 (F: 5′-AGGAGGGGATCCGCGGCGGCGGCGGCGAC- 3′ and R: 5′-GTTAAGCTTTTAGGTGAATATCTGGCGTAG-3′), which contained *Bam*H I and *Hin*d III sites (underlined), respectively. The PCR products were cloned into *Bam*H I -*Hin*d III-digested pET-28a(+) to obtain pET-28a(+)-mTaHO1 by using the *N*-terminal 6× *his*-tag. The integrity of the construct was verified by restriction analysis and complete DNA sequencing of the insert (GenScript, Nanjing, China). The constructed vector was transferred into *Escherichia coli* strain BL21 (DE3). The mTaHO1 protein expression was induced by 1 mM isopropyl β-d-thiogalactopyranoside (IPTG) at 28 °C for 12 h, based on the manufacturer’s instructions (Novagen), and purified through Ni-NTA column and Sephadex G-25 chromatography. After centrifugation at 20,000 g in a rotor (model Avanti J-25, Beckman) for 30 min, the purified mTaHO1 protein was used for the following biochemical experiments.

### 3.5. HO Activity Determination

HO activity was analyzed using the method described in our previous reports [14,21,35]. In the HO activity test, the concentration of biliverdin IX (BV) was estimated using a molar absorption coefficient at 650 nm of 6.25 mM^−1^·cm^−1^ in 0.1 M HEPES-NaOH buffer (pH 7.2). One unit of activity (U) was calculated by taking the quantity of enzyme to produce 1 nmol BV per 30 min. Protein concentration was determined according to [36] using bovine serum albumin as the standard. *K*_m_ and *V*_max_ values were calculated for mTaHO1 using Lineweaver-Burk plot. For hemin, the data were obtained using the standard assay, with the concentrations varied between 1 and 20 μM. The effects of temperature and pH were determined using the standard assay conditions as described above.

### 3.6. Subcellular Localization of TaHO1

The coding region for the putative transit peptide for TaHO1 was amplified from wheat cDNA with the following primer pairs P3 (F: 5′-GCCGACTAGTCCGCGACCATCCTC-3′ and R: 5′-CTCGGTACCCGGGGATCCTCTAGA-3′) containing *Spe* I and *Nco* I sites (underlined), respectively, and was cloned into *Spe* I-*Nco* I-digested pCAMBIA 1302 to generate an in-frame fusion with the green fluorescent protein (GFP) reporter gene under the control of CaMV 35S promoter. The transient assays of fluorescent protein expression in Nicotiana benthamiana were performed according to [37] by using Agrobacterium tumefaciens EHA105. Preparation of bacterium and tobacco seedlings, and tobacco inoculation were carried out as described by [38]. After 2 d of co-cultivation, the signals for GFP and chloroplast autofluorescence were examined under a TCS-SP2 confocal laser scanning microscope (Leica Lasertechnik GmbH, Heidelberg, Germany).

### 3.7. Gel Blot Analysis for TaHO1

Primary antibody used was rabbit polyclonal antibody raised against recombinant mTaHO1 with a molecular mass of 31 kD. Sixty μg of protein from crude membrane fraction was subjected to SDS-PAGE using a 12.5% acrylamide resolving gel [21]. Separated proteins were then transferred to polyvinylidene difluoride (PVDF) membranes, and non-specific binding of antibodies was blocked with 5% non-fat dried milk in phosphate-buffered saline (PBS, pH 7.4) for 2 h at room temperature. Membranes were then incubated overnight at 4 °C with primary antibodies diluted 1:2000 in PBS buffer plus 1% non-fat dried milk. Immune complexes were detected using horseradish peroxidase (HRP)-conjugated goat anti-rabbit immunoglobulin G. The color was developed with a solution containing 3,3′-diaminobenzidine tetrahydrochloride (DAB) as the HRP substrate. Coomassie Brilliant Blue-stained gel was present to show that equal amounts of protein was loaded. Additionally, the films were scanned (Uniscan B700^+^, Tsinghua Unigroup Ltd., Beijing, China) and analysed using Quantity One v4.4.0 software (Bio-Rad, USA).

### 3.8. Real-Time RT-PCR Analysis

Real-time quantification RT-PCR reactions were performed in a Mastercycler^®^ ep *realplex* real-time PCR system (Eppendorf, Hamburg, Germany) using the SYBR^®^ *Premix Ex Taq*™ (TaKaRa Bio Inc., Dalian, China) according to the manufacturer’s instructions. The PCR reaction was performed using the following primers: for *TaHO1* (GenBank ID: HM014348), P4 (F: 5′-AATACTGGGTTGGAGAGA-3′ and R: 5′-AGAAGTGGCAAATAAATG-3′); and for *actin* (GenBank ID: AB181991), P5 (F: 5′-TCTGGTGATGGTGTGAGC-3′ and R: 5′-CGGTTGTTGTGAGGGAGT-3′). Gene-specific primers were designed with the software tool Primer Express (Applied Biosystems, Foster city, CA, USA). All reactions were set up in triplicate. Relative expression levels are presented as values relative to corresponding control sample at the indicated times, after normalization to *actin* transcript levels.
